# Response-metrics for acute lung inflammation pattern by cobalt-based nanoparticles

**DOI:** 10.1186/s12989-015-0089-1

**Published:** 2015-05-13

**Authors:** Jiyoung Jeong, Youngju Han, Craig A. Poland, Wan-Seob Cho

**Affiliations:** Lab of Toxicology, Department of Medicinal Biotechnology, College of Health Sciences, Dong-A University, 840 Hadan-2dong, Saha-gu, Busan, 604-714 Republic of Korea; Institute of Occupational Medicine, Research Avenue North, Riccarton, Edinburgh, UK

**Keywords:** Dose-metric, High solubility nanoparticles, Ion metric, Co_3_O_4_, CoO, Intratracheal instillation

## Abstract

**Background:**

Although the surface area metric has been proposed as a possible dose-metric for nanoparticles (NPs), it is limited to low-solubility NPs and the dose-metric for high-solubility NPs is poorly understood. In this study, we aimed to assess the appropriate dose-metric or response-metric for NPs using two cobalt (Co)-based NPs, cobalt monoxide (CoO) and cobalt oxide (Co_3_O_4_), which both show distinctive solubility, and determine the role of their soluble Co ions in inflammation.

**Methods:**

We evaluated the physicochemical properties of NPs, including solubility in artificial lysosomal fluid (ALF, pH 5.5). Acute lung inflammogenicity was evaluated by bronchoalveolar lavage fluid analysis using the rat intratracheal instillation model. The appropriate response-metric was then determined by plotting several dose-metrics against parameters for lung inflammation. To investigate the effect of the soluble fraction of CoO NPs, the equivalent doses of Co ions from CoCl_2_ were instilled.

**Results:**

The Co_3_O_4_ and CoO NPs showed about 11.46 % and 92.65 % solubility in ALF, respectively. Instillation of Co_3_O_4_ NPs produced neutrophilic inflammation, but CoO NPs induced eosinophilic inflammation. The number of eosinophils showed good correlation with the soluble Co ions dose from NPs (r^2^ = 0.987, *p* <0.001), while the number of neutrophils showed good correlation with the surface area dose of the biopersistent NPs (r^2^ = 0.876, *p* <0.001). Instillation of CoCl_2_ showed a similar type and magnitude of inflammation as CoO NPs.

**Conclusions:**

In the Co-based NPs, the eosinophilic inflammation was produced by Co ions based on the ion metric, while the neutrophilic inflammation was developed based on the surface area metric of the biopersistent NPs.

## Background

The selection of an appropriate dose-metric for nanomaterials (NMs) is important for evaluating their toxicity and assessing their risk [1]. Mass, surface area (SA), and number are the possible candidates for dose-metrics for the particulate form of NMs, while the number of fibers of a specific length is an additional candidate for the dose-metric of high aspect ratio NMs (HARNs) [2, 3]. Most studies used lung inflammation models (generally by intratracheal instillation), because the lung is very sensitive to both acute and chronic inflammation and is essentially a closed chamber with very few fraction of the instilled NPs that translocate into the systemic circulation [4]. For example, instillation of gold NPs larger than 5 nm diameter showed less than 1 % extrapulmonary translocation [4]. In an intratracheal instillation model, the SA metric for low-solubility NPs has been proposed as a better metric than either mass or number [5–7]. Under a SA metric, the number of neutrophils of low-solubility low-toxicity NPs showed an overlapped dose–response curve, while intrinsic factors such as surface reactivity contribute to the slope of the curves for low-solubility high-toxicity NPs [5, 7].

Although NPs are rarely soluble in normal physiological environments such as in the interstitial fluid, some NPs such as zinc oxide (ZnO), copper oxide (CuO), and silver (Ag) have considerable solubility in acidic environments such as lysosomal and gastric fluid [8–10]. High-solubility NPs composed of toxic elements generally show higher toxicity than low-solubility NPs of the same compositional elements via the Trojan-horse type mechanism of toxicity [11]. In recent studies, high-solubility ZnO, CuO, and Ag NPs showed acute eosinophilic inflammation with increased cytotoxicity due to their solubilized compositional ions in the rat intratracheal instillation model, while medium-solubility nickel oxide (NiO) and cobalt oxide (Co_3_O_4_) NPs caused acute neutrophilic inflammation with immunological responses in the chronic phase, such as delayed type hypersensitivity [8, 12, 13]. Therefore, the evaluation of the differential effects of soluble fraction and biopersistent NPs, using NPs with the same compositional elements but different solubility, might provide important information on the role of solubility in NP toxicology.

Unlike low-solubility NPs, the dose-metric for high-solubility NPs is poorly understood. The first in evaluating the dose-metrics for high-solubility NPs could be the comparison of acute lung inflammogenicity using two NPs with the same compositional elements but different solubility. The next step would then be to compare the dose–response relationship between the high solubility NPs (e.g., Ag, CuO, and ZnO) by normalizing the observed values with the weighed toxicity value of the solubilized ions (e.g., AgCl_2_, CuCl_2_, and ZnCl_2_). In this study, we obtained two cobalt-based NPs, cobalt monoxide (CoO) and cobalt oxide (Co_3_O_4_), having a distinct difference in solubility in artificial lysosomal fluid. We then evaluated the role of soluble fraction and biopersistent NPs in acute lung inflammation, using an intratracheal instillation model, to investigate the appropriate response-metric for acute lung inflammation by cobalt-based NPs.

## Results

### Characterization of NPs

**Fig. 1:**
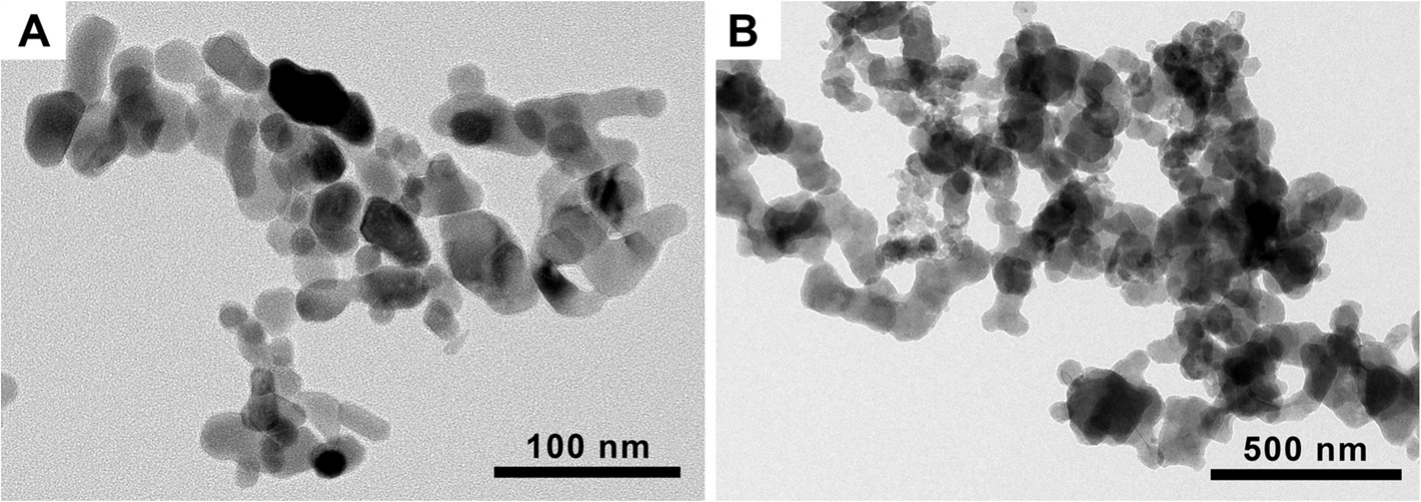
Transmission electron microscopy (TEM) image of (**A**) cobalt oxide (Co_3_O_4_) and (**B**) cobalt monoxide (CoO) nanoparticles (NPs). Both Co_3_O_4_ and CoO NPs showed spherical shape without porous structure

The physicochemical properties of NPs are presented in Table 1. The average size of Co_3_O_4_ and CoO NPs measured by transmission electron microscopy (TEM) were 20.2 and 65.4 nm, respectively. TEM images showed that both NPs were spherical without porous structure (Fig. 1). The hydrodynamic size of NPs showed that both NPs showed a “hard agglomerates” in both distilled water (DW) and phosphate-buffered saline (PBS). Co_3_O_4_ NPs were less agglomerated than CoO NPs when dispersed in DW, while both NPs showed similar size range about 450 nm when dispersed in PBS (Table 1). Polydispersity showed that Co_3_O_4_ NPs were more homogenous than CoO NPs. The zeta potentials of both NPs were positive in DW but negative in PBS, which may be due to the neutral pH of PBS. Incubation of Co_3_O_4_ and CoO NPs in artificial lysosomal fluid (ALF) showed 11.46 % and 92.65 % solubility, respectively. Solubility of NPs in PBS was minimal at 0.02 % and 4.12 % for Co_3_O_4_ and CoO NPs, respectively. Both NPs showed no endotoxin contamination.

**Table 1:** Physicochemical characterization of nanoparticles

Measure	Co_3_O_4_	CoO
Primary size (nm)	20.2 ± 0.4	65.4 ± 2.8
Hydrodynamic size (nm)		
in DW	93.4 ± 1.8	380.3 ± 14.4
in PBS	468.9 ± 24.3	449.1 ± 8.9
Polydispersity		
in DW	0.18 ± 0.03	0.72 ± 0.02
in PBS	0.26 ± 0.02	0.69 ± 0.05
Surface area (m^2^/g)	35.8	8.5
Zeta potential (mV)		
in DW	39.53 ± 0.72	6.99 ± 1.18
in PBS (pH 7.4)	−26.40 ± 0.90	−21.80 ± 0.45
Solubility (%) in ALF	11.46	92.65
Solubility (%) in PBS	0.02	4.12
Endotoxin	ND	ND

Data were presented as mean ± SEM

ND, not detected

### Cytological evaluation of lung inflammogenicity

Instillation of Co_3_O_4_ NPs at the high dose produced significant increases in the total number of cells (45.60 ± 7.71, × 10^5^) and neutrophils (25.65 ± 4.80, ×10^5^) compared to the vehicle control, but the number of macrophages, eosinophils, and lymphocytes were comparable to vehicle control (Table 2). Treatment of CoO NPs significantly increased the number of macrophages at the mid dose and eosinophils (3.37 ± 1.23, ×10^5^) at the high dose (Table 2).

**Table 2:** Summary of inflammatory cell counts, LDH, total protein, and inflammatory mediators at 24 h after intratracheal instillation of NPs

BALF measure	VEH_PBS_	Co_3_O_4_ dose (μg/rat)			CoO dose (μg/rat)		
		40	100	400	40	100	400
Total cells (× 10^5^)	10.95 ± 1.42	23.60 ± 3.64	20.48 ± 5.59	45.60 ± 7.71^***^	18.90 ± 2.40	30.53 ± 0.39	23.25 ± 4.10
Macrophages (× 10^5^)	10.34 ± 1.19	23.21 ± 3.55	16.67 ± 4.63	19.82 ± 3.27	17.58 ± 1.81	24.46 ± 2.64^*^	11.83 ± 3.03
Neutrophils (× 10^5^)	0.49 ± 0.39	0.38 ± 0.10	3.79 ± 3.13	25.65 ± 4.80^***^	1.18 ± 1.06	4.91 ± 2.02	8.02 ± 1.85
Eosinophils (× 10^5^)	0.07 ± 0.02	0.00 ± 0.00	0.01 ± 0.01	0.09 ± 0.05	0.10 ± 0.10	1.16 ± 0.76	3.37 ± 1.23^**^
Lymphocytes (× 10^5^)	0.05 ± 0.05	0.02 ± 0.02	0.00 ± 0.00	0.05 ± 0.05	0.03 ± 0.03	0.00 ± 0.00	0.04 ± 0.04
LDH (fold vs control)	1.00 ± 0.05	1.23 ± 0.10	1.62 ± 0.17	3.47 ± 0.15^***^	2.35 ± 0.08^***^	5.41 ± 0.22^***^	9.21 ± 0.30^***^
Total protein (μg/mL)	301 ± 5	118 ± 14	335 ± 116	253 ± 18	99 ± 5	1104 ± 77^*^	3767 ± 667^***^
CINC-3 (pg/mL)	0.00 ± 0.00	0.00 ± 0.00	0.00 ± 0.00	110.4 ± 39.4^**^	0.00 ± 0.00	27.3 ± 27.3	0.00 ± 0.00
IL-6 (pg/mL)	0.00 ± 0.00	0.00 ± 0.00	0.00 ± 0.00	0.00 ± 0.00	0.00 ± 0.00	123.4 ± 43.6	982.0 ± 349.4^***^
Eotaxin (pg/mL)	5.7 ± 1.2	4.5 ± 1.5	67.4 ± 57.4	22.5 ± 10.6	4.5 ± 1.8	265.7 ± 68.9	2963.9 ± 870.7^***^
IL-13 (pg/mL)	17.7 ± 1.9	22.6 ± 4.2	28.9 ± 4.5	16.9 ± 6.0	35.8 ± 4.9	49.0 ± 9.7	108.5 ± 15.6^***^

Data were presented as mean ± SEM. ^*^ *p* < 0.05, ^**^ *p* < 0.01, and ^***^ *p* < 0.001, statistically different from vehicle control (VEH_PBS_)

VEH_PBS_, Ca^2+^-and Mg^2+^-free PBS was used as VEH for NPs; LDH, lactate dehydrogenase; CINC-3, cytokine-induced neutrophil chemoattractant-3

### Lactate dehydrogenase (LDH) and total protein

The levels of LDH by Co_3_O_4_ NPs were significantly increased only at high dose (400 μg/rat). However, the levels of LDH in CoO NPs were significantly increased in all treatment groups in a dose-dependent manner (Table 2). The fold increase of LDH in CoO NPs (9.21 ± 0.30) at 400 μg/rat showed much higher level than Co_3_O_4_ NPs (3.47 ± 0.15) at the same dose (Table 2). The concentrations of total protein were significantly increased by the mid- and high-dose group of CoO NPs, while Co_3_O_4_ NPs showed no significant increases (Table 2).

### Expression of pro-inflammatory cytokines in bronchoalveolar lavage fluid (BALF)

Among the 6 pro-inflammatory cytokines measured in BALF, treatment with Co_3_O_4_ NPs significantly increased cytokine-induced neutrophil chemoattractant-3 (CINC-3) levels only, while CoO NPs significantly increased the levels of interleukin-6 (IL-6), eotaxin, and IL-13 in a dose-dependent manner (Table 2). IL-1β and tumor necrosis factor-α (TNF-α) showed no significant changes in either treatment groups (data not shown).

### Effects of dissolved Co ions on eosinophil recruitment by CoO NPs

**Fig. 2:**
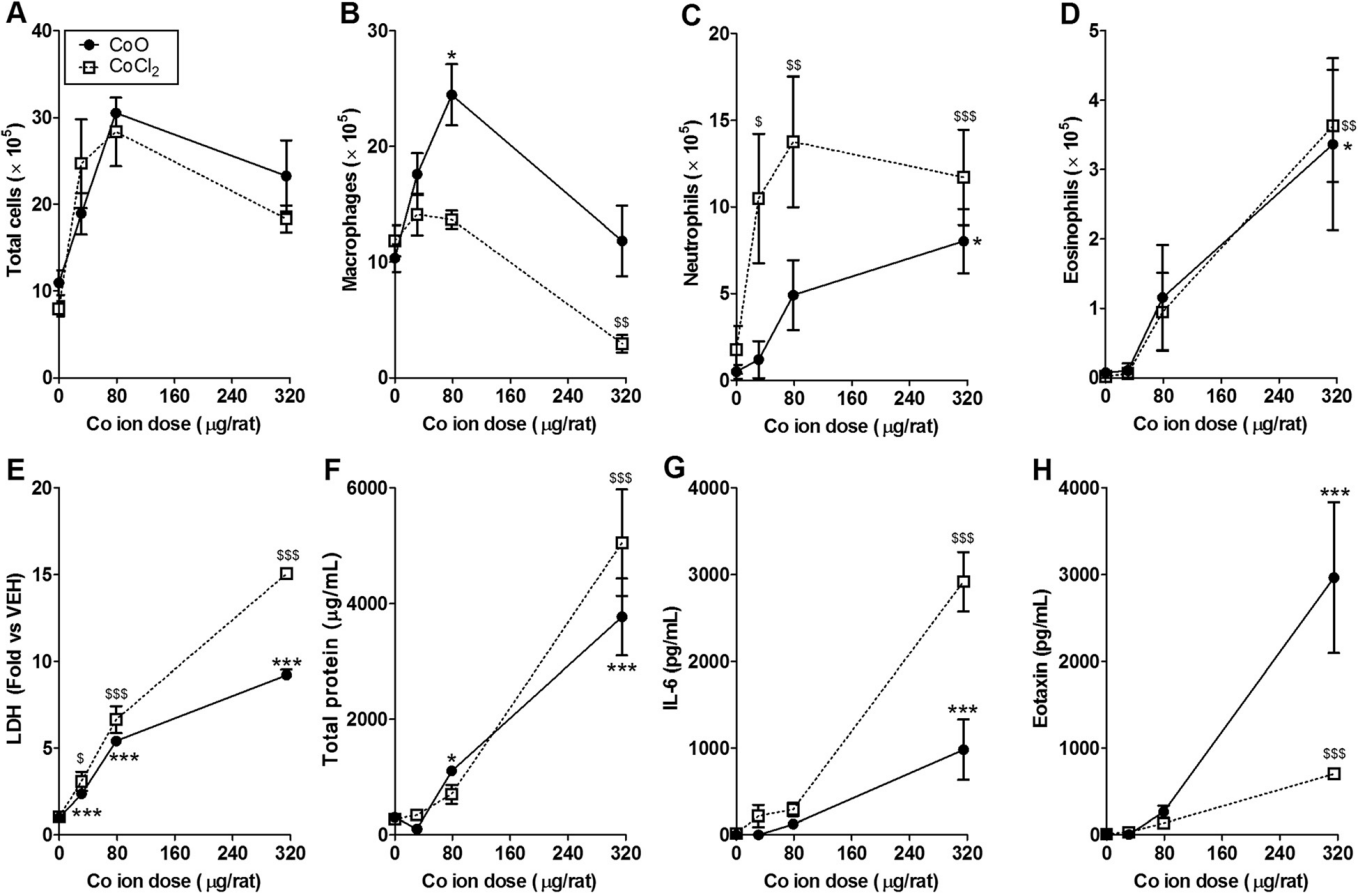
Comparison of inflammatory parameters for cobalt monoxide (CoO) nanoparticles (NPs) and cobalt chloride (CoCl_2_). Number of (**A**) total cells, (**B**) macrophages, (**C**) neutrophils, (**D**) eosinophils, and levels of (**E**) LDH, (**F**) total protein, (**G**) IL-6, and (**H**) eotaxin. Both treatment groups had same doses for cobalt ions (31, 79, and 315 μg/rat). Note that the inflammatory parameters of CoO NPs were similar with the CoCl_2_ which implies that the toxicity of CoO NPs was derived from their solubilized cobalt ions. Values are mean ± SEM (*n* = 4) for each treatment group. The data from CoO NPs were compared with the vehicle control (Ca^2+^- and Mg^2+^-free phosphate buffered saline) to determine statistical significance. ^*^ *p* < 0.05, ^**^ *p* < 0.01, ^***^ *p* < 0.001. The data from CoCl_2_ were compared with the vehicle control (0.9 % saline). ^$^ *p* < 0.05, ^$$^ *p* < 0.01, ^$$$^ *p* < 0.001

To evaluate the effect of dissolved Co ions, we instilled cobalt chloride (CoCl_2_) dissolved in sterile saline into the lungs of rats at an equivalent Co ion dose for CoO NPs. Treatment of CoCl_2_ showed significant increases in the number of neutrophils and eosinophils, the levels of LDH and total protein, and the levels of IL-6 and eotaxin in BALF compared to the vehicle control (Table 3). Based on the equivalent dose of Co ions (31, 79, and 315 μg/rat), the data from CoCl_2_ produced similar dose–response curves compared to the CoO NPs in various inflammatory markers including the inflammatory cells, LDH, total protein, IL-6, and eotaxin (Fig. 2).

**Table 3:** Summary of inflammatory cell counts, LDH, total protein, and inflammatory mediators at 24 h after intratracheal instillation of CoCl_2_

BALF measure	VEH_saline_	CoCl_2_ (μg/rat)^a^		
		69 (31)^b^	173 (79)	693 (315)
Total cells (× 10^5^)	13.65 ± 2.63	24.68 ± 5.12	28.35 ± 3.96	18.30 ± 1.54
Macrophages (× 10^5^)	11.84 ± 1.36	14.12 ± 1.81	13.66 ± 0.80	2.92 ± 0.746^**^
Neutrophils (× 10^5^)	1.76 ± 1.38	10.48 ± 3.72^*^	13.74 ± 3.77^**^	11.70 ± 2.75^***^
Eosinophils (× 10^5^)	0.02 ± 0.02	0.06 ± 0.04	0.95 ± 0.56	3.63 ± 0.81^**^
Lymphocytes (× 10^5^)	0.05 ± 0.01	0.10 ± 0.04	0.04 ± 0.02	0.02 ± 0.02
LDH (fold vs control)	1.00 ± 0.12	3.08 ± 0.53^*^	6.65 ± 0.78^***^	15.05 ± 0.18^***^
Total protein (μg/mL)	270 ± 47	341 ± 67	700 ± 165	5051 ± 917^***^
CINC-3 (pg/mL)	0.00 ± 0.00	0.00 ± 0.00	0.00 ± 0.00	0.00 ± 0.00
IL-6 (pg/mL)	0.00 ± 0.00	0.00 ± 0.00	39.2 ± 39.2	734.6 ± 152.3^***^
Eotaxin (pg/mL)	10.76 ± 3.27	30.94 ± 16.60	133.1 ± 49.2	699.8 ± 38.1^***^
IL-13 (pg/mL)	0.00 ± 0.00	0.00 ± 0.00	0.00 ± 0.00	0.00 ± 0.00

^a^Co ions were prepared by dissolving CoCl_2_ in saline and treated at equivalent dose for Co of CoO NPs (40, 100, and 400 μg/rat)

^b^Parenthesis represents the Co ions mass for CoCl_2_ which is equivalent Co ions doses of CoO NPs

Data were presented as mean ± SEM. ^*^ *p* < 0.05, ^**^ *p* < 0.01, and ^***^ *p* < 0.001, statistically different from vehicle control (VEH_saline_)

VEH_saline_, sterile 0.9 % saline was used as a vehicle control for CoCl_2_; LDH, lactate dehydrogenase; CINC-3, cytokine-induced neutrophil chemoattractant-3

### Correlation between dose-metrics and inflammatory parameters

**Fig. 3:**
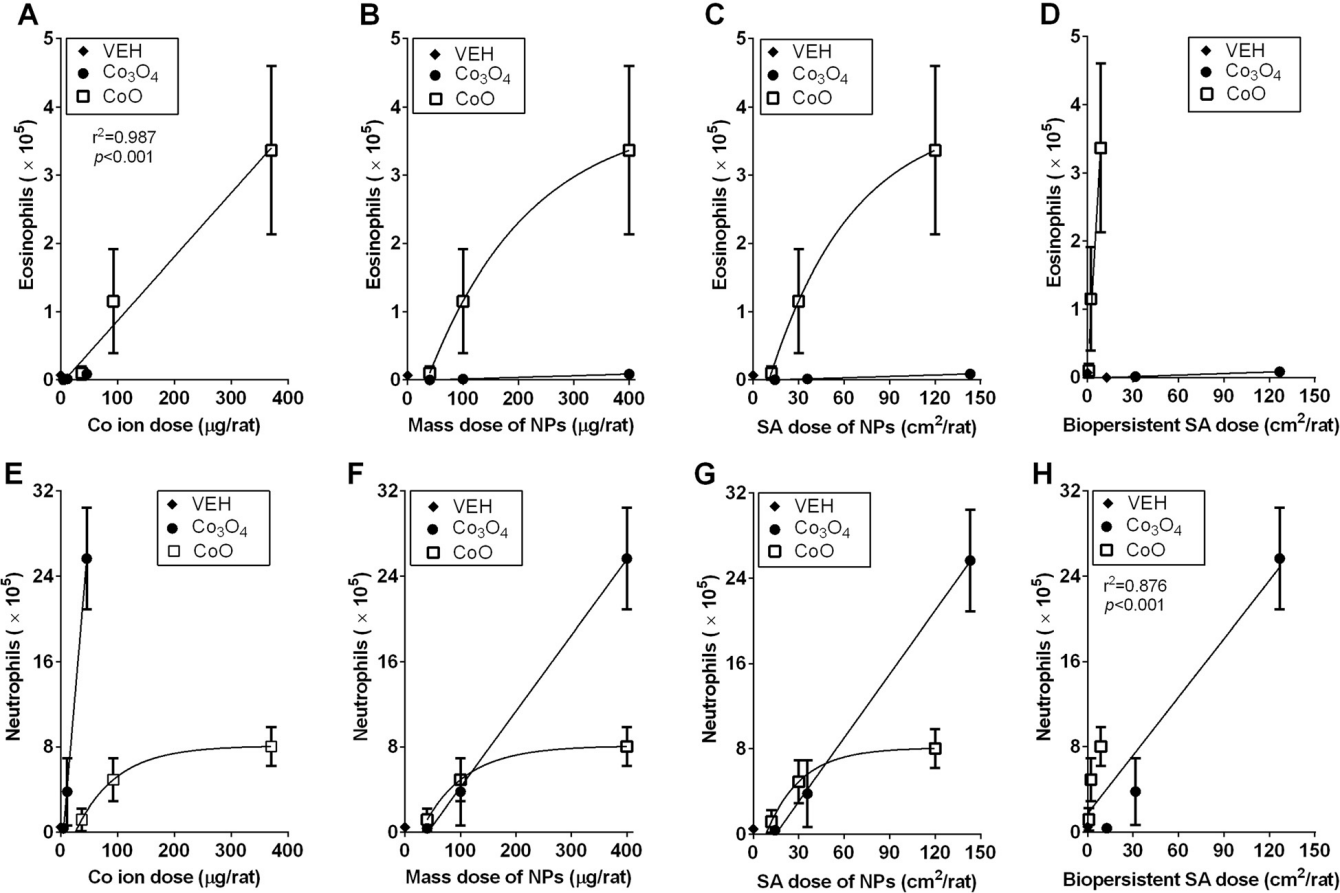
Correlation between the inflammatory parameters of nanoparticles (NPs) and dose-metrics including soluble Co ions, mass, surface area (SA), and SA dose of biopersistent NPs. Correlation between the number of eosinophils and (**A**) Co ions, (**B**) mass dose of NPs, (**C**) SA dose of NPs, or (**D**) SA dose of biopersistent NPs. Correlation between the number of neutrophils and (**E**) Co ions, (**F**) mass dose of NPs, (**G**) SA dose of NPs, or (**H**) SA dose of biopersistent NPs. When the dose–response curves of cobalt oxide (Co_3_O_4_) and cobalt monoxide (CoO) NPs were significantly different, separate curves were drawn with either linear regression or non-linear regression, while when the dose–response curves of each NP overlapped, the combined curves were prepared with best-fit regression models and the Pearson correlation test was applied. Values are mean ± SEM (*n* = 4) for each treatment group

**Fig. 4:**
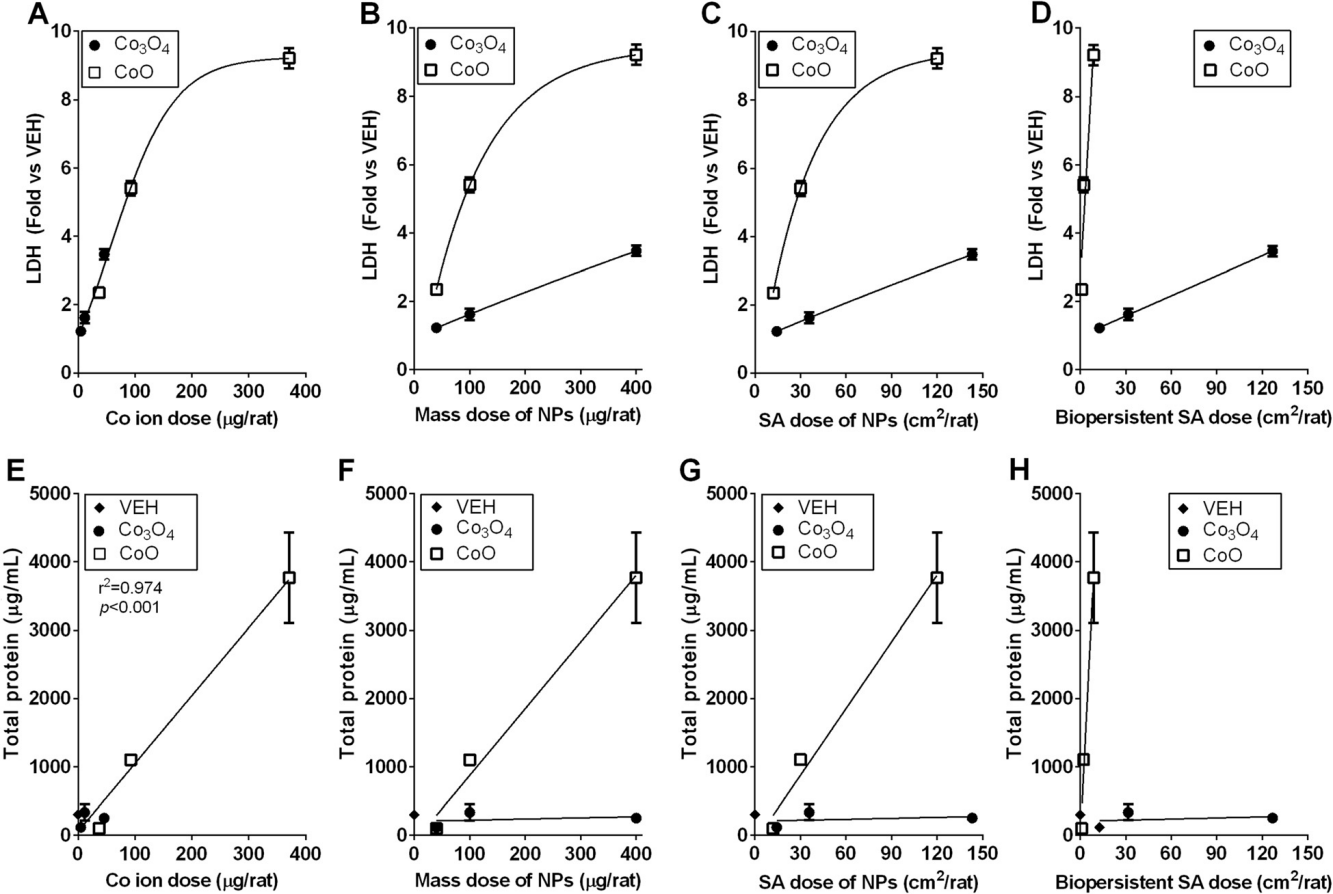
Correlation between the toxic parameters [the levels of lactate dehydrogenase (LDH) and total protein] of nanoparticles (NPs) and dose-metrics, including soluble cobalt (Co) ions, mass, surface area (SA), and SA dose of biopersistent NPs. Correlation between the levels of LDH and (**A**) Co ions, (**B**) mass dose of NPs, (**C**) SA dose of NPs, or (**D**) SA dose of biopersistent NPs. Correlation between the levels of total protein and (**E**) Co ions, (**F**) mass dose of NPs, (**G**) SA dose of NPs, or (**H**) SA dose of biopersistent NPs. When the dose–response curves of cobalt oxide (Co_3_O_4_) and cobalt monoxide (CoO) NPs were significantly different, separate curves were drawn with either linear regression or non-linear regression, while when the dose–response curves of each NP overlapped, the combined curves were prepared with best-fit regression models, and the Pearson correlation test was applied. Values are mean ± SEM (*n* = 4) for each treatment group

To evaluate the best-fit dose–response curve, various dose-metrics, including mass, SA, fraction of soluble Co ions, and SA of biopersistent NPs, were plotted against the acute lung inflammatory parameters produced by Co_3_O_4_ and CoO treatment. When the number of eosinophils from each NP was plotted against mass, SA, or SA of biopersistent NPs, each NP showed a separate dose–response curve, while the dose–response curves from two NPs overlapped when they were plotted against soluble Co ions (r^2^ = 0.987, *p* < 0.001) (Fig. 3A-D). When the number of neutrophils from each NP was plotted against various dose-metrics, only the SA dose of biopersistent NPs showed good correlation (r^2^ = 0.876, *p* < 0.001), while other dose-metrics showed separate curves (Fig. 3E-H). In line with the dose–response curves of eosinophils, the dose–response curves of LDH and total protein from NPs overlapped when they were plotted against the soluble Co ion dose, while other dose-metrics showed separate curves (Fig. 4).

## Discussion

Selection of an appropriate dose-metric for NMs is critical for the evaluation of toxicity and risk assessment. The dose-metric for NMs is intricately related to the factors that produce the response. Therefore, the dose-metric of NMs can be variable depending on the biologically effective dose (BED), which is the component of the total dose that produces adverse effects [2]. The BED is influenced not only by the intrinsic characteristics of NMs, including size, shape, soluble fraction, and biopersistence, but also by the extrinsic factors including route of exposure. Among them, the inhalation route is well studied, and SA and number of fibers with specific length were proposed as a proper dose-metric for NPs and fibers, respectively [2]. Although the SA metric has been proposed as a dose-metric for NPs, it is limited to low-solubility NPs such as titanium dioxide and carbon black [5, 6]. Based on the SA dose-metric, the slope of the dose–response curve of low-solubility NPs is dependent on the surface reactivity, such as zeta potential and reactive oxygen species generation potential [7, 8]. However, the dose-metric for high-solubility NPs was poorly understood, although the soluble fraction was known as one of the main factors that influence the BED [14, 15].

Most NPs are rarely soluble at normal physiological conditions, but solubility can be accelerated in acidic conditions, such as in the lysosomal fluid inside cells [16, 17]. Therefore, the significant solubility of NPs in ALF shown in this study is solely due to the chemical reaction. However, further study is needed for the development of a biopersistence assay using a cell-based model or *in vivo* condition because the biodegradation of NPs can also be accelerated by the enzyme-catalyzed reaction [18, 19]. The dissolution of NPs results in the loss of their surface reactivity, and thus the factors inducing toxicity may depend on the toxicity of their composition ions. Therefore, the SA metric cannot be applied to high-solubility NPs and another proper dose-metric should be considered. Moreover, because the toxicity of high-solubility NPs such as zinc oxide (ZnO) and copper oxide (CuO) were known to cause higher inflammogenicity and cytotoxicity in the acute phase with severe eosinophilic inflammation, a proper dose-metric is very important for risk assessment [20]. Here, we used Co_3_O_4_ and CoO, two types of cobalt-based NPs, which have similar types of soluble fractions but different solubility, to evaluate the dose-metric or response-metric for acute lung inflammogenicity.

The Occupational Safety and Health Administration (OSHA) regulated the 8-h permissible exposure limit (PEL) for cobalt metal, dust, and fumes as 0.1 mg/m^3^ [21]. The retained mass of 100 μg in the lung, the mid dose in this study, would be reached from 28 days (6 h/day) of nasal exposure to a 0.1 mg/m^3^ cloud of NPs with aerodynamic diameter of 50 nm (typical primary size of NPs used in this study) according to the Multiple-Path Particle Dosimetry (MPPD) deposition model [22]. In our previous study, we found that high solubility NPs can produce acute eosinophilic inflammation but the dose was critical for determining the type of inflammation [8]. Acute eosinophilic inflammation was also reported in the case of an accidental exposure of ZnO to human [23]. Although the bolus deliveries of NPs at a high dose which can be reached after a repeated inhalation scenario do not reflect physiological condition, the high dose used in this study (400 μg/rat) can be a realistic dose for occupational exposure.

In this study, medium-solubility Co_3_O_4_ NPs produced a dose-dependent neutrophilic influx, while high-solubility CoO NPs induced an eosinophilic influx into the lung 24 h after treatment. In our previous studies, intratracheal instillation of high-solubility NPs such as ZnO and CuO NPs produced an acute eosinophilia in the lung without any previous sensitization [16, 20, 24]. In this study, the inflammatory potentials of CoO NPs, evaluated by counting cells or measuring cytokines in the BALF, were comparable to ones evaluated after treatment with an identical Co ion mass of CoCl_2_, although there was some variation in the number of macrophages and the levels of LDH, IL-6, and eotaxin. Therefore, the eosinophilic inflammation produced by CoO NPs was originated from the dissolution of Co ions present inside lysosomes, which is the same mechanism of action as ZnO NPs [16, 25]. This eosinophilic inflammation was mediated by direct induction of eotaxin but the underlying mechanisms were poorly understood. One possible mechanism for non-IgE mediated allergic response is the activation of anaphylatoxins such as C3a and C5a which was reported in some liposomal NPs (e.g., Doxil) and micellar NPs (e.g., Taxol) [26]. However, that mechanism was not known for the acute eosinophilic inflammation by metal oxide NPs which warrants further investigation.

In previous studies, SA has been proposed as a dose-metric for low-solubility NPs in the acute pulmonary inflammation model [5, 27, 28]. For nanofibers, the number of fibers with a specific length has been proposed as one of the main factors contributing to lung inflammation [29–31]. However, the dose-metric for high-solubility NPs has not been reported yet. According to the results from this study, the ion dose-metric can be used for eosinophilic inflammation, cell death, and vascular permeability, because the number of eosinophils and levels of LDH or total protein produced by Co_3_O_4_ and CoO NPs had an overlapping dose–response curve when it was plotted against the dose of Co ions. In addition, the SA dose-metric of biopersistent NPs can be used for neutrophilic inflammation because the number of neutrophils produced by Co_3_O_4_ and CoO NPs showed a significant correlation when plotted against the SA of biopersistent NPs, while other dose-metrics showed distinctive graphs. These results also imply that the soluble fraction of NPs is the main contributor of eosinophilic inflammation, cell death, and vascular permeability, while the SA of biopersistent NPs is the main contributor of neutrophilic inflammation.

## Conclusions

Instillation of two types of Co-based NPs produced different types of inflammation, neutrophilic or eosinophilic inflammation for Co_3_O_4_ and CoO NPs, respectively. The eosinophilic inflammation was produced by the dissolved Co ions inside of cells, and thus the appropriate dose-metric for eosinophil influx, cell death, and vascular permeability is the ion metric. On the other hand, neutrophilic inflammation was developed due to the role of biopersistent NPs, and the appropriate dose-metric for neutrophil influx should be the SA metric of biopersistent NPs.

## Methods

### NPs and physicochemical characterization

Co_3_O_4_ and CoO NPs were selected as cobalt-based NPs to evaluate both the role of solubility for NPs and dose-metric for high-solubility NPs. Both NPs were purchased from Nanostructured & Amorphous Materials Inc., (NanoAmor; Houston, TX, USA). The primary size of the NPs was measured by TEM (JEM-1200EX II, JEOL, Tokyo, Japan). The SA of Co_3_O_4_ NPs was measured by ParticlesCIC Ltd. (Leeds, UK) using a Micromeritics Tristar 3000 analyzer (Micromeritics Ltd., Bedfordshire, UK) and the SA of CoO NPs was measured by Center for Collaborative Instruments at Ulsan National Institute of Science and Technology (Ulsan, Korea) using a ASAP 2420 (Micromertics Ltd.). The average size was obtained by measuring at least 100 particles using a built-in image analyzer program (JEOL). The hydrodynamic size, polydispersity, and zeta potential of NPs in DW or PBS was measured using a Zetasizer-Nano ZS (Malvern, Malvern Hills, UK). The levels of endotoxin were measured in NPs dispersed in sterile PBS at 800 μg/mL, which is equivalent to 400 μg/rat, the highest dose for an animal study, using an endpoint chromogenic *Limulus* Amebocyte lysate (LAL) assay kit (Cambrex, Walkersville, MD, USA). The detection limit of the LAL kit was 0.1 EU/mL.

### NP solubility test

Evaluation of solubility is essential to understand the role of soluble fractions in NP toxicity. The solubility is variable depending on their environment. Therefore, in our study we incubated NPs with either ALF (55 mM NaCl, 150 mM NaOH, 108 mM citric acid, 0.87 mM CaCl_2_, 0.67 mM Na_2_HPO_4_ · 7H_2_O, 0.27 mM Na_2_SO_4_, 0.52 mM MgCl_2_ · 6H_2_O, 0.64 mM glycerin, 0.26 mM sodium citrate dihydrate, 0.39 mM sodium tartrate dihydrate, 0.76 mM sodium lactate, 0.78 mM sodium pyruvate, 1 mL of formaldehyde, pH 5.5) [19] or PBS, pH 7.4 at 100 μg/mL for 24 h at room temperature. After incubation, NP-free supernatant was collected using three rounds of centrifugation at 15,000 *g* for 30 min. The concentration of soluble cobalt ions was measured by the Center for Collaborative Instruments at Dong-A University (Busan, Korea) using inductively coupled mass spectrometry (ICP-MS) (Hewlett-Packard 4500; Yokogawa, Japan). The fraction of solubilized cobalt ions was calculated and expressed as a percentage by dividing the mass of cobalt ions by the initial mass of cobalt in Co_3_O_4_ or CoO NPs.

### Intratracheal instillation of NPs

The intratracheal instillation model was selected instead of an inhalation study because the former is an easy and reliable method to identify NP toxicity and compare responses to different particle types [32]. NP suspensions at 80, 200, and 800 μg/mL were prepared by dispersing NPs in sterile Ca^2+^- and Mg^2+^-free PBS (Life Technologies, Gaithersburg, MD, USA) and sonicated for 10 min using a bath sonicator (Saehan-Sonic, Seoul, Korea) to break up agglomerates. NP suspensions were prepared and sonicated immediately before use as recommend in a previous study [33]. Six-week-old specific-pathogen-free female rats were purchased from Samtako (Gyeonggi-do, Korea), maintained, and handled according to the policies approved by the Institutional Animal Care and Use Committee of Dong-A University. The instillation (*n* = 4 per group) was performed according to a previously described method [24]. NP suspensions were instilled at doses of 40, 100, and 400 μg/rat by instilling 0.5 mL of the suspensions.

### Preparation of BALF

Twenty-four hour post-instillation, the rats were euthanized via an intraperitoneal injection of tiletamine-zolazepam (Zoletil®, 50 mg/kg) and xylazine (5 mg/kg). The trachea was cannulated with a blunt 14 gauge needle, and the lungs were lavaged *in situ* four times with cold sterile Ca^2+^- and Mg^2+^-free PBS (Life Technologies) at a volume of 8 mL. The first lavage was kept for analysis of LDH, total protein, and pro-inflammatory cytokines. Cell pellets from four lavages were pooled for cell counts. The total number of cells in the BALF was quantified by a nucleocounter (Chemometec, Allerod, Denmark), and 4 × 10^4^ cells were attached to glass slides by cytospin at 27 *g* for 5 min. The cells were then fixed for 5 min with methanol and stained with Diff-Quik (Thermo Fisher Scientific, Waltham, MA, USA) for the differential cell count.

### Analysis of BALF

To evaluate the inflammogenicity using cytological analysis, differential cell counts were performed by counting a minimum of 300 cells under a light microscope (Nikon, Tokyo, Japan). The levels of LDH, a marker for cytotoxicity, were measured in the BALF using an LDH assay kit (Roche Diagnostics, Mannheim, Germany). The concentrations of total protein, a maker for vascular permeability, were measured in the BALF using a bicinchoninic acid (BCA) assay kit (Thermo Fisher Scientific). To evaluate the role of pro-inflammatory cytokines in NP-induced inflammation, CINC-3, eotaxin, IL-1β, IL-6, IL-13, and TNF-α, all from the Duoset ELISA kit (R&D Systems, Minneapolis, MN, USA), were measured in the BALF according to the manufacturer’s protocol.

### Instillation of Co ions into the lungs of rats

In our previous study, we found that the eosinophilic inflammation by ZnO NPs was attributed to its high solubility in lysosomal fluid [16]. Because CoO NPs showed a high solubility in ALF and significantly recruited eosinophils, CoCl_2_ was used to further investigate the role of the dissolved Co ions from CoO NPs. CoCl_2_ (Sigma-Aldrich, St. Louis, MO, USA) was solubilized in a sterile solution of 0.9 % saline, because Co ions can easily precipitate in a phosphate solution by forming cobalt phosphate, Co_3_(PO_4_)_2_, which is insoluble in DW. The concentrations of CoCl_2_ for instillation were 69, 173, and 693 μg/rat, which were the equivalent Co ion doses for 40, 100, and 400 μg/rat of CoO NPs, respectively. As a vehicle control, 500 μL of saline was instilled.

### Evaluation of appropriate dose-metric

There are many metrics should be regarded for dose–response curves. To our knowledge, dose can be divided into treatment-related dose (mass, number, or SA) and particle-derived dose (surface charge, oxidative potential, solubility, or soluble toxins). In this study, we used Co_3_O_4_ and CoO, two types of cobalt-based NPs, which have similar types of soluble fractions but different solubility to evaluate the dose-metric or response metric for acute lung inflammation pattern. Regarding the treatment-related dose, number of particles was excluded because particle number is the worst to describe NP-induced pulmonary inflammatory effects [6]. Regarding the particle-derived dose, surface charge and oxidative potential was excluded not only because we focused on the solubility issue but also the surface charge or oxidative potential is a moving target for high solubility NPs. For this reason, we treated two-cobalt based NPs with a mass metric and converted to several possible dose metrics such as mass of NPs, SA of NPs, soluble fraction of Co ions, or SA of biopersistent NPs. The SA of biopersistent NPs was mathematically calculated by multiplying the ratio of dissolution by the SA of instilled NPs. The appropriate dose-metrics were then plotted against the parameters for lung inflammogenicity, including total cells, differential cell counts, LDH, total protein, and pro-inflammatory cytokines. When the dose–response curves of Co_3_O_4_ and CoO NPs were significantly different, separate curves were drawn with either linear regression or non-linear regression using GraphPad Prism Software (GraphPad Prism version 6 for Windows; GraphPad Software, Inc., San Diego, CA, USA). However, when the dose–response curve of each NP was overlapped, combined curves were prepared with best-fit regression models, and the Pearson correlation test was applied.

### Statistical analysis

Data were expressed as mean ± SEM (*n* = 4). We analyzed statistical differences using one-way analysis of variance (ANOVA) followed by post-hoc Tukey’s pairwise comparisons using GraphPad Prism Software (GraphPad Prism version 6 for Windows). The value of *p* < 0.05 considered statistically significant.

## References

[1] Simko M, Nosske D, Kreyling WG (2014). Metrics, dose, and dose concept: the need for a proper dose concept in the risk assessment of nanoparticles. Int J Environ Res Public Health.

[2] Donaldson K, Schinwald A, Murphy F, Cho WS, Duffin R, Tran L (2013). The biologically effective dose in inhalation nanotoxicology. Acc Chem Res.

[3] Wittmaack K (2007). In search of the most relevant parameter for quantifying lung inflammatory response to nanoparticle exposure: particle number, surface area, or what?. Environ Health Perspect.

[4] Kreyling WG, Hirn S, Moller W, Schleh C, Wenk A, Celik G (2014). Air-blood barrier translocation of tracheally instilled gold nanoparticles inversely depends on particle size. ACS Nano.

[5] Duffin R, Tran L, Brown D, Stone V, Donaldson K (2007). Proinflammogenic effects of low-toxicity and metal nanoparticles in vivo and in vitro: highlighting the role of particle surface area and surface reactivity. Inhal Toxicol.

[6] Oberdorster G, Oberdorster E, Oberdorster J (2007). Concepts of nanoparticle dose metric and response metric. Environ Health Perspect.

[7] Rushton EK, Jiang J, Leonard SS, Eberly S, Castranova V, Biswas P (2010). Concept of assessing nanoparticle hazards considering nanoparticle dosemetric and chemical/biological response metrics. J Toxicol Environ Health A.

[8] Cho WS, Duffin R, Thielbeer F, Bradley M, Megson IL, Macnee W (2012). Zeta potential and solubility to toxic ions as mechanisms of lung inflammation caused by metal/metal oxide nanoparticles. Toxicol Sci.

[9] Cho WS, Kang BC, Lee JK, Jeong J, Che JH, Seok SH (2013). Comparative absorption, distribution, and excretion of titanium dioxide and zinc oxide nanoparticles after repeated oral administration. Part Fibre Toxicol.

[10] Setyawati MI, Yuan X, Xie J, Leong DT (2014). The influence of lysosomal stability of silver nanomaterials on their toxicity to human cells. Biomaterials.

[11] Ortega R, Bresson C, Darolles C, Gautier C, Roudeau S, Perrin L (2014). Low-solubility particles and a Trojan-horse type mechanism of toxicity: the case of cobalt oxide on human lung cells. Part Fibre Toxicol.

[12] Cho WS, Duffin R, Bradley M, Megson IL, MacNee W, Howie SEM (2012). NiO and Co_3_O_4_ nanoparticles induce lung DTH-like responses and alveolar lipoproteinosis. Eur Respir J.

[13] Silva RM, Xu J, Saiki C, Anderson DS, Franzi LM, Vulpe CD (2014). Short versus long silver nanowires: a comparison of in vivo pulmonary effects post instillation. Part Fibre Toxicol.

[14] Nel AE, Madler L, Velegol D, Xia T, Hoek EM, Somasundaran P (2009). Understanding biophysicochemical interactions at the nano-bio interface. Nat Mater.

[15] Braakhuis HM, Park MV, Gosens I, De Jong WH, Cassee FR (2014). Physicochemical characteristics of nanomaterials that affect pulmonary inflammation. Part Fibre Toxicol.

[16] Cho WS, Duffin R, Howie SE, Scotton CJ, Wallace WA, Macnee W (2011). Progressive severe lung injury by zinc oxide nanoparticles; the role of Zn2+ dissolution inside lysosomes. Part Fibre Toxicol.

[17] Adamcakova-Dodd A, Stebounova LV, Kim JS, Vorrink SU, Ault AP, O′Shaughnessy PT (2014). Toxicity assessment of zinc oxide nanoparticles using sub-acute and sub-chronic murine inhalation models. Part Fibre Toxicol.

[18] Kotchey GP, Hasan SA, Kapralov AA, Ha SH, Kim K, Shvedova AA (2012). A natural vanishing act: the enzyme-catalyzed degradation of carbon nanomaterials. Acc Chem Res.

[19] Stopford W, Turner J, Cappellini D, Brock T (2003). Bioaccessibility testing of cobalt compounds. J Environ Monit.

[20] Cho WS, Duffin R, Poland CA, Duschl A, Oostingh GJ, Macnee W (2012). Differential pro-inflammatory effects of metal oxide nanoparticles and their soluble ions in vitro and in vivo; zinc and copper nanoparticles, but not their ions, recruit eosinophils to the lungs. Nanotoxicology.

[21] 29 CFR1910.1000 Table Z-1, Occupational Safety and Health Regulations (OSHA Standards).

[22] Cassee FR, Muijser H, Duistermaat E, Freijer JJ, Geerse KB, Marijnissen JC (2002). Particle size-dependent total mass deposition in lungs determines inhalation toxicity of cadmium chloride aerosols in rats. Application of a multiple path dosimetry model. Arch Toxicol.

[23] Castet D, Bouillard J (1992). Acute pneumopathy caused by exposure to zinc oxide. Rev Mal Respir.

[24] Cho WS, Duffin R, Poland CA, Howie SE, MacNee W, Bradley M (2010). Metal oxide nanoparticles induce unique inflammatory footprints in the lung: important implications for nanoparticle testing. Environ Health Perspect.

[25] Proper SP, Saini Y, Greenwood KK, Bramble LA, Downing NJ, Harkema JR (2014). Loss of hypoxia-inducible factor 2 alpha in the lung alveolar epithelium of mice leads to enhanced eosinophilic inflammation in cobalt-induced lung injury. Toxicol Sci.

[26] Szebeni J (2014). Complement activation-related pseudoallergy: a stress reaction in blood triggered by nanomedicines and biologicals. Mol Immunol.

[27] Monteiller C, Tran L, MacNee W, Faux S, Jones A, Miller B (2007). The pro-inflammatory effects of low-toxicity low-solubility particles, nanoparticles and fine particles, on epithelial cells in vitro: the role of surface area. Occup Environ Med.

[28] Oberdorster G, Oberdorster E, Oberdorster J (2005). Nanotoxicology: an emerging discipline evolving from studies of ultrafine particles. Environ Health Perspect.

[29] Donaldson K, Tran CL (2004). An introduction to the short-term toxicology of respirable industrial fibres. Mutat Res.

[30] Schinwald A, Chernova T, Donaldson K (2012). Use of silver nanowires to determine thresholds for fibre length-dependent pulmonary inflammation and inhibition of macrophage migration in vitro. Part Fibre Toxicol.

[31] Schinwald A, Murphy FA, Prina-Mello A, Poland CA, Byrne F, Movia D (2012). The threshold length for fiber-induced acute pleural inflammation: shedding light on the early events in asbestos-induced mesothelioma. Toxicol Sci.

[32] Driscoll KE, Costa DL, Hatch G, Henderson R, Oberdorster G, Salem H (2000). Intratracheal instillation as an exposure technique for the evaluation of respiratory tract toxicity: uses and limitations. Toxicol Sci.

[33] Lison D, Laloy J, Corazzari I, Muller J, Rabolli V, Panin N (2009). Sintered indium-tin-oxide (ITO) particles: a new pneumotoxic entity. Toxicol Sci.

